# Socio-demographic determinants of the severity of locomotor disability among adults in Bangladesh: a cross-sectional study, December 2010–February 2011

**DOI:** 10.1186/s13690-017-0217-5

**Published:** 2017-11-20

**Authors:** Ilias Mahmud, Lynda Clarke, George B. Ploubidis

**Affiliations:** 1 James P Grant School of Public Health, BRAC University, 5th Floor icddr, b Building, 68 Shahid Tajuddin Ahmed Sharani Mohakhali, Dhaka, 1212 Bangladesh; 2 London School of Hygiene and Tropical Medicine, Keppel Street, London, UK; 3 Centre for Longitudinal Studies, Department of Social Science, University College London, 55 – 59 Gordon Square, London, WC1H 0NU UK

**Keywords:** Disability, Locomotor disability, Physical disability, Bangladesh, Determinants

## Abstract

**Background:**

Socio-demographic variables are widely known to have an association with the presence of any disability. However, the association between the severity of locomotor disability and socio-demographic variables has never been investigated in Bangladesh.

**Methods:**

A cross sectional survey of adults with locomotor disabilities was conducted between December 2010 and February 2011 at the Centre for the Rehabilitation of the Paralysed (CRP), Dhaka, Bangladesh. During the study period 328 adults with locomotor disabilities met our selection criteria, but 316 consented and participated in the study. The 55-item Locomotor Disability Scale was used to measure disability. This study investigated the socio-demographic determinants of the severity of locomotor disability: age, gender, marital status, educational attainment, occupation, income status, type of house, living in own/rented house, household monthly income, household population and area of residence.

**Results:**

Participants’ age was positively associated with the severity of their locomotor disability (β = 0.01; 95% CI: 0.004 to 0.02), adjusting for diagnosis and other socio-demographic variables studied. Individuals who had an income experienced 0.35 (95% CI: -0.63 to −0.07) points decrease in the severity of disability than those did not have an income, adjusting for diagnosis and rest of the socio-demographic variables studied. In comparison to the unemployed individuals, students, homemakers, and individuals in elementary occupation respectively experienced 0.75 (95% CI: -1.08 to −0.43), 0.51 (95% CI: -0.82 to −0.19) and 0.37 (95% CI: -0.66 to −0.08) points decrease in the severity of locomotor disability, adjusting for diagnosis and rest of the socio-demographic variables studied.

**Conclusions:**

The severity of locomotor disability has an association with individuals’ age, income status and occupation of the adults with such disability in Bangladesh. No such association was evident with other socioeconomic position and demographic variables. This finding suggests that people with locomotor disabilities in Bangladesh experience similar disabling built and attitudinal environments irrespective of their socioeconomic positions and demographic characteristics. Further community-based studies are needed to confirm such conclusions.

**Electronic supplementary material:**

The online version of this article (doi:10.1186/s13690-017-0217-5) contains supplementary material, which is available to authorized users.

## Background

Disability is an evolving concept. Over the past decades there has been a paradigm shift from a medical model towards a more social model of disability. This paradigm shift was evident with the introduction of the International Classification of Functioning, Disability and Health (ICF) in 2001 [1]. According to the ICF, disability does not result from individuals’ health conditions alone, but when the negative aspects of their health conditions (impairments) are confronted with unfavourable environmental (physical, social and attitudinal) and personal factors [1]. The inclusion of such contextual factors in the ICF framework validates their role as the important determinants of disability.

There is growing evidence of the association between individuals’ societal conditions and their health [2–4]; and of a strong relationship between socio-economic position and the incidence of disabling health conditions [5, 6]. Interaction between an unfavourable social context and impairment reduces capabilities, and restricts the functioning of people with disabilities (PWDs), Amartya Sen argued [7]. Likewise, social theories of disability suggest that disability is largely determined by social determinants, the conditions in which people are born, grow, live, work and age [5, 8].

Among the social determinants of health, socioeconomic position has received particular attention from social researchers [3, 9–11]. Income, education and occupation have frequently been used as socioeconomic position measures [3, 11]. Literature suggests that a higher level of income is associated with better access to health and social services, better diet, better housing and working conditions, and a lower level of exposure to environmental pollutants, among others [3, 12–14]. Education is another powerful indicator of socioeconomic position. There is ample evidence of a strong association between education and lifestyle, risk behaviours and the prevention of ill health, problem solving ability related to health, and social values, such as individuals’ perceived importance of looking after their own health [3]. Occupation has been found to be associated with differential exposures to physical and psychological risk factors, individuals’ ability to obtain good housing, and access to health care, among others [3]. The effects of socioeconomic position on health have been widely investigated in high-income countries, but less work has been done on this topic in low-income countries [3]. To date, there has been no investigation of the association between socio-demographic characteristics and the severity of locomotor disability (LD) in Bangladesh.

The higher estimated prevalence of disability among adults in low-income countries (18%) compared to high-income countries (11.8%) [15] perhaps is a testimony to the role of socio-economic determinants of disability. Globally, there are inequalities in health, both between and within countries, and these inequalities are largely avoidable [2, 16]. Likewise, there are avoidable inequalities in living a disability free life, both between and within countries. There is growing research evidence suggesting that many of these health inequalities are due to social factors [2]. Amartya Sen argued that poor and disadvantaged groups, including PWDs, should have greater access to public goods and services in order to achieve equality in their capabilities [17]. Given this scenario, this study investigated whether socio-demographic variables are associated with the severity of LD among adults in Bangladesh.

## Methods

A cross sectional survey of adults with locomotor disabilities was conducted between December 2010 and February 2011 at the Centre for the Rehabilitation of the Paralysed (CRP), Dhaka, Bangladesh. The CRP is a non-governmental, not for profit, specialised rehabilitation centre in Bangladesh. It provides holistic rehabilitation services to people with locomotor disabilities. CRP offers both institutional-based (in-patient, out-patient and vocational) and community-based rehabilitation (CBR) services which are managed by a multidisciplinary team involving medical doctors, nurses, physiotherapists, occupational therapists, speech and language therapists, trained staff in orthotics and prosthetics, counsellors, vocational trainers and social workers [18].

### Sampling

**Fig. 1:**
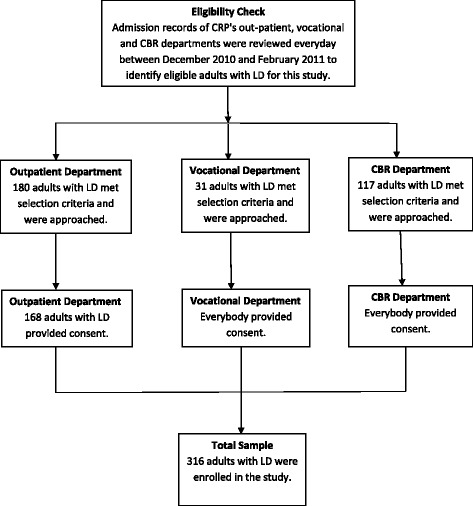
Flowchart of the sampling design, cross-sectional survey of adults with locomotor disabilities, Centre for the Rehabilitation of the Paralysed, Bangladesh, December 2010–February 2011

**Fig. 2:**
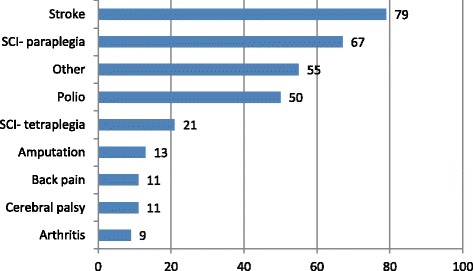
Clinical characteristics of the participants- a cross sectional survey of adults with locomotor disabilities, Centre for the Rehabilitation of the Paralysed, Dhaka, Bangladesh, December 2010–February 2011

Participants were recruited from the out-patient, vocational and CBR departments of the CRP. Adults with locomotor disabilities were considered eligible for this study if they were living in a community at the time of interview for at least the past 30 consecutive days; aged between 18 and 65 years; and who did not have any of the following co-morbidities: cognitive and perceptual problems, dementia or problems with memory, psychiatric disorders, or any other medical emergency. Participants’ diagnoses were checked from the medical notes of the CRP. During the study period 328 adults with locomotor disabilities met our selection criteria and 316 consented and participated in the study. Out of the total 316 participants, 199 were recruited from CRP’s out-patient and vocational training departments; and were interviewed at the centre. The remaining 117 participants were recruited through CRP’s CBR programme and were interviewed in their own community. A flowchart of the sampling design is provided as Fig. 1. Characteristics of the participants are presented in Table 1 and Fig. 2.

**Table 1:** Socio-demographic characteristics of the participants- a cross sectional survey of adults with locomotor disabilities, Centre for the Rehabilitation of the Paralysed, Dhaka, Bangladesh, December 2010–February 2011

Characteristics	Mean (SD)/Median/Proportions (95% CI)
Age (in years)	
Mean (SD)	37.3 (13.8)
Gender	
Male	67.7% (62.3–72.7%)
Female	32.3% (27.3–37.7%)
Marital status	
Currently married	59.4% (53.8–64.7%)
Divorced/separated/widowed	5.7% (3.6–8.9%)
Never married	34.9% (29.8–40.4%)
Educational attainment	
Less than primary	23.4% (19.1–28.4%)
Primary	33.5% (28.5–39.0%)
Lower Secondary	14.2% (10.8–18.6%)
Upper Secondary or higher	28.8% (24.0–34.1%)
Occupation	
Unemployed	44.6% (39.2–50.2%)
Elementary	12.7% (9.4–16.8%)
Public/private service	13.0% (9.7–17.2%)
Business	10.4% (7.5–14.4%)
Student	8.2% (5.7–11.8%)
Housewife	11.1% (8.0–15.1%)
Monthly income status	
Earning	37% (31.8–42.5%)
Median (those earning)	BDT 6000 (74.4 USD)
Housing condition-type of house	
Non-brick built house	59.3% (53.8–64.7%)
Flat	12.4% (9.2–16.5%)
Brick built house	28.3% (23.5–33.5%)
Area of residence- Rural	66.4% (60.9–71.4%)
Household monthly income	
Median	BDT 9750 (120.9 USD)
Min-Max	BDT 0.00 - 80,000.00 (0–992.2 USD)
Household population	
Mean (SD)	4.8 persons (2.13)

### Data collection

Four data collectors and the first author conducted all interviews using a structured questionnaire. All data collectors were university graduate and had previous data collection experience in disability studies. Among them, two had social science background and were also involved in a previous qualitative study conducted to develop the Locomotor Disability Scale (LDS); one was an occupational therapy graduate and the other was a physiotherapy graduate. All of the data collectors received 5 days training on data collection. Training was conducted by the first author and an experienced occupational therapist working in Bangladesh.

### Disability measurement

Participants’ disability was measured using the LDS. The LDS is a 55-item ordinal scale. Its items include mobility activities and activities from all aspects of occupational performance: activities of daily living, productivity/work and leisure. It asks the respondents to rate the difficulty they face in performing each of the selected activity items. The LDS response options ranges from 0 to 4, where ‘0′ indicates no difficulty and ‘4′ indicates extreme difficulty or inability to perform the activity. The LDS items were developed through qualitative research (which yielded 70 activity items) [19]; and were refined using exploratory and confirmatory factor analysis (which retained 55 activity items). Factor analysis confirmed a one-factor model meaning all 55 item measure only one underlying construct- locomotor disability. Further detail of the development of the LDS is discussed elsewhere [20]. Total LD score for each individual was calculated by the application of the latent variable model, where high score indicates severe disability. In order to derive the total LD score, the Full Information Maximum Likelihood (FIML) was used to deal with missing values. The 55-item LDS is presented as Additional file 1: Table S1.

### Assessment of socio-demographic status

1. Demographic variables included age, gender, marital status, and area of residence. Area of residence was categorised as rural or urban according to the definition used in the Bangladesh Demographic and Health Surveys [21, 22].
2. Classic measures of socioeconomic position [3, 23]:
  - Income: respondents’ household monthly income data were used. Income was reported in BDT (Bangladesh currency). Household members included those who share food from the same pot.
  - Education: the International Standard Classification of Education (ISCED) [24] was used to define educational attainment of the participants.
  - Occupation: If participants were involved in more than one occupation at the time of interview, their main occupation was recorded. Participants’ occupations were grouped under the following categories: unemployed, elementary occupation, public/private service, business, student and homemaker. Unemployed group included those without a paid or un-paid job at the time of interview. Elementary occupation included agricultural labours, transport workers, labours in office and other industries and other elementary workers [25]. Students included those who were continuing their formal study by any means, either attending an institution or at their own home with a special arrangement with their educational institution. Homemakers included housewives who were actively performing all or some of their household responsibilities at the time of the interview. Housewives who could not perform their household work and were not involved in any other paid or un-paid job and were not studying in a formal institution were grouped as unemployed.
3. Other socioeconomic position variables included ‘type of house’ and a dichotomous variable, indicating whether participants were living in their own house or in rented house at the time of the interview. The participants’ home was recorded as ‘flat’ if they were living in a set of rooms which are part of a larger building; ‘brick house’ if they were living in a brick built detached or semi-detached building and as ‘non-brick house if they were living in a mud, bamboo, bush and/or tin made house.

### Statistical analyses

For the descriptive analysis, mean and standard deviation or median and range were reported for continuous variables; and categorical variables were reported as proportions (Table 1). Linear regression analysis was performed to estimate regression coefficients relating LD for the selected explanatory variables. The continuous latent trait disability scores were used in linear regression analyses. Both bivariate and multivariable linear regression analyses were performed. The Stata 13 data analysis and statistical software was used for data analyses.

## Results

### Characteristics of the study participants

The study participants are described in Table 1 and Fig. 2. They were aged between 18 and 65 years with a mean of 37.3 years (Standard Deviation, SD = 13.8). The majority (59.4%, *n* = 187) of them were married and rural residents (66.4%, *n* = 209), and two-thirds (67.7%, *n* = 214) of them were male. Over a fifth (23.4%, *n* = 74) of the participants had less than primary education; just over one-third (33.5%, *n* = 106) had primary education; and over a quarter (28.8%, *n* = 91) had upper secondary or higher educational attainment. The majority (63%, *n* = 199) of the participants did not have any income. The median monthly household income was BDT 9750 (USD 120.9) ranging from BDT 0 to 80,000. One tenth (10.1%, *n* = 31) of the households did not have any income at all. On average, each household had 4.8 members with a SD of 2.13. Participants had a wide range of disabling conditions including spinal cord injury, stroke, arthritis, chronic back pain, cerebral palsy, poliomyelitis and amputation (Fig. 2). Participants’ minimum and maximum disability scores were −1.4 and 3.3, respectively; while the mean score was 0.2 (95% CI: 0.1–0.3).

### Socio-demographic predictors of severity of locomotor disability

In bivariate linear regression analyses, presented in Table 2, evidence was found for an association between the severity of locomotor disability (LD) and age; marital status; educational attainment; and occupation type; and income status of the participants. However, no evidence was found for an association between the severity of LD and gender; household income; type of house, living in own home; household population size; and area of residence.

**Table 2:** Multivariable linear regression model predicting severity of disability, cross-sectional survey of adults with locomotor disabilities, Centre for the Rehabilitation of the Paralysed, Bangladesh, December 2010–February 2011

Variables	Bivariate analyses		Multivariable model (R^2^ = 0.43; adj R^2^ = 0.37)	
	β	95% CI	β	95% CI
Age	.02	.01 to.02	.01	.004 to .02
Gender				
Male	0		0	
Female	−.13	−.32 to .05	.09	−.12 to .29
Marital status				
Currently married	0		0	
Divorced/separated/widowed	.48	.10 to .86	.30	−.04 to .64
Never married	−.24	−.43 to −.06	.12	−.11 to .34
Educational attainment				
Less than primary	0		0	
Primary	.02	−.25 to .22	.10	−.11 to .31
Lower secondary	.20	−.49 to .10	.15	−.10 to .41
Upper secondary or higher	−.25	−.49 to −.005	.11	−.13 to .36
Occupation				
Unemployed	0		0	
Elementary	−.52	−.77 to −.27	−.37	−.66 to −.08
Public/private service	−.63	−.88 to −.38	−.14	−.52 to .23
Business	−.84	−1.12 to −.57	−.30	−.66 to .07
Student	−1.01	−1.31 to −.71	−.75	−1.08 to −.43
Homemaker	−.62	−.89 to −.36	−.51	−.82 to −.19
Income status- Earning				
No	0		0	
Yes	−.44	−.61 to −.26	−.35	−.63 to −.07
Type of house				
Non-brick house	0		0	
Flat	−.02	−.30 to .25	.11	−.22 to .44
Brick house	−.03	−.24 to .17	−.02	−.21 to .18
Living in own house	.10	−.11 to .30	.13	−.11 to .37
Household monthly income (thousand BDT)	−.004	−.01 to .002	−.004	−.01 to .002
Household population	.01	−.03 to .05	−.01	−.05 to .003
Residence-Rural	0		0	
Urban	−.01	−.19 to .18	.13	−.10 to .36
Diagnosis				
SCI-tetraplegia	0		0	
SCI- paraplegia	−.59	−.93 to −.25	−.51	−.85 to −.18
Stroke	−.53	−.87 to −.19	−.71	−1.04 to −.37
Arthritis	−1.10	−1.64 to −.55	−1.08	−1.60 to −.56
Chronic back pain	−1.31	−1.82 to −.80	−1.20	−1.70 to −.71
Cerebral palsy	−1.26	−1.77 to −.76	−1.00	−1.50 to −.52
Polio	−1.47	−1.83 to −1.12	−1.20	−1.55 to −.85
Amputation	−.99	−1.47 to −.51	−.80	−1.25 to −.34
Other	−.97	−1.32 to −.62	−.96	−1.30 to −.62

A year increase in age was associated with a 0.02 (95% CI:0.01 to 0.02) point increase in locomotor disability. Being divorced, separated or widowed was associated with a 0.48 (95% CI:0.10 to 0.86) point increase in the severity of LD when compared to being currently married. On the other hand, being never married was associated with 0.24 (95% CI:−0.43 to −0.06) points decrease in LD when compared to being currently married. Upper secondary or higher educational attainment was associated with 0.24 (95% CI:−0.49 to −0.005) points decrease in LD in comparison to less than primary educational attainment. In comparison to unemployed individuals, individuals in elementary occupation, public/private service, business, students and homemakers were respectively experienced 0.52 (95% CI:−0.77 to −0.27), 0.63 (95% CI:−0.88 to −0.38), 0.84 (95% CI: −1.12 to −0.57), 1.01 (95% CI:−1.31 to −0.71) and 0.62 (95% CI:−0.89 to −0.36) points decrease in the severity of LD.

In multivariable linear regression analysis, after controlling for diagnosis, evidence for an association was found only between the severity of LD and participants’ age; income status; and occupation type (Table 2). No evidence of association, after controlling for diagnoses, was found between the severity of LD and gender; marital status; educational attainment; household monthly income; type of house; living in own home; household population size; and area of residence. Participants’ age was positively associated with the severity of their LD with 1 year increase in age was associated with 0.01 points increase in the severity of LD (95% CI: 0.01 to 0.03), adjusting for diagnosis and rest of the socio-demographic variables. In comparison to the unemployed individuals, individuals in elementary occupation, students and homemakers experienced 0.37 (95% CI: -0.66 to −0.08), 0.75 (95% CI: -1.08 to −.43) and 0.51 (95% CI: -0.82 to −0.19) points decrease in the severity of LD, respectively after adjusting for diagnosis and rest of the socio-demographic variables. However, no statistically significant differences in the severity of LD were observed between unemployed individuals and individuals employed in public/private services; and individuals running their own business. On the other hand, individuals with an income experienced 0.35 (95% CI: -0.63 to −0.07) points decrease in the severity of LD when compared to individuals without any income, adjusting for diagnosis and rest of the socio-demographic variables. This multivariable linear regression model predicting the severity of LD among different socio-demographic groups, after adjusting for diagnoses accounted for 37% of the total variation in LD (Table 2).

## Discussion

This study investigated the socio-demographic determinants of the severity of LD: age, gender, marital status, educational attainment, occupation, income status, type of house, living in own/rented house, household monthly income, household population and area of residence, adjusting for diagnosis. Statistically significant evidence of association was found between the severity of LD and age; occupation types; and income status of the adults with LD.

Age is recognised as a major determinant of disability [3, 26]. Risk of severe disabling conditions increases with age [27]. On the other hand, some disabling conditions are progressive, such as osteoarthritis [28]; hence, severity of these conditions increases with age. In addition, older people are more likely to suffer from age related co-morbidities independent to their disabling conditions [29], which might compromise their ability to cope with their disability. In this study, we observed that the severity of LD increased with age. Similarly, another survey of adults aged 60 years and older in 535 villages in Bangladesh found that reporting of severe disabilities increased with age [30]. The Bangladesh Household Income and Expenditure Survey 2010 too found that the proportion of people experience disability increases with age [26].

We found that respondents’ type of occupation has an association with the severity of their LD. Individuals in elementary occupation, students and homemakers experienced significantly lesser degrees of LD than unemployed individuals. However, no such differences were observed between individuals employed in public/private sectors and individuals running their own business, and unemployed individuals. PWDs experience inaccessibility and attitudinal barriers in Bangladesh [31]. Therefore, mostly people with mild impairments and/ or people who could adapt their job tasks and environment to fit them could continue their occupation. Similar evidence has also been found elsewhere in the world [3]. Homemakers/housewives (housewives who could not perform household tasks were coded as unemployed) and students have more control over the nature of their job and environment. In addition, they have less involvement with the outside world. Perhaps, therefore, they experience less severe forms of LD than other occupation groups. Whereas, self-employed and employed people need to frequently encounter the outside world; have little control over their job tasks and environment; therefore, experiencing severe forms of LD. Those with the most severe LDs are unable to continue any job; and thus become unemployed (unemployment resulting from the disability rather than preceding it). A study of PWDs, who successfully completed vocational training from a rehabilitation centre in Bangladesh, identified inaccessible built environment and public transport as the prime inhibiting factors for re-employment of PWDs in Bangladesh [32]. Other barriers of re-employment of PWDs were physical impairment, lack of motivation, and family and community members’ negative attitudes towards PWDs [32].

Disability prevalence is higher among women in Bangladesh [26, 30]. However, no previous studies in Bangladesh have investigated the association between the severity of LD and gender. Nevertheless, this study found no evidence to suggest an association between the severity of LD and gender. There is gender inequality in terms of accessing health and other services in Bangladesh [33]; which, perhaps, explains the higher disability prevalence among women in Bangladesh. In this study participants were recruited from a rehabilitation centre, so women of this study had already overcome the gender barrier to access rehabilitation services. These women were receiving the same rehabilitation services; and living in a similar socio-political and geographical context as the men of this study. Hence, these environments would probably be equally disabling for both men and women. However, if this study could include the women who had not overcome the gender barriers in accessing rehabilitation services, it might have found some association between gender and the severity of LD.

Bivariate linear regression analysis provided evidence of association between the severity of LD and marital status. However, in the adjusted model no evidence was found. Perhaps, age worked as a confounding variable in this regard; since divorced, separated or widowed participants were older and never married participants were younger compared to the currently married participants.

A previous disability survey in Bangladesh found that the likelihood of having any disability decreases with education [34]. However, our study found lack of evidence to suggest that educational attainment is associated with the severity of LD. Education increases individuals’ knowledge and access to information, but the severity of disability may be largely determined by other contextual factors (such as inaccessibility of physical spaces, transport and inappropriate rules and legislations for PWDs). Higher education might increase compliance to the rehabilitation programme, thus highly educated people might develop better capacity than those with poor educational attainment following rehabilitation. However, modifications to the external environment are beyond any individual’s control, but subject to the societal and governmental influences and interventions. Therefore, educational attainment might have an association with the ‘capacity’ of PWDs (performance after neutralising the impact of the societal context) [1], but not with their ‘performance’ (executing activities in a real life situation) [1].

Income is widely known to have an association with disability [3, 34]. Impoverished people are more likely to have a disability, and PWDs are more likely to become poor losing their income opportunities [35], and managing treatment and rehabilitation costs. However, in our study, no evidence was found to suggest an association between household monthly income and the severity of LD. The Government of Bangladesh does not take responsibility for the home modifications of disabled people. Perhaps their income, even the income of the highest quartile, was not enough to undertake necessary environmental modifications in their homes in order to maximise their functional independence. The very low wages for informal domestic workers in Bangladesh [36] could be another reason for not finding any difference in the severity of disability by household income. Employing somebody or bringing a distant poor family member into the household to look after the disabled member is more economical than bringing in environmental modifications.

We did not find any evidence to suggest an association between the severity of LD and the housing condition; and living in own or rented house. Poor socioeconomic position is widely known to have an association with poor health outcomes and disability [3]. However, perhaps once a person become disabled in Bangladesh, the severity of their disability cannot be determined by their housing conditions. Better quality brick built houses/flats and poor quality non-brick houses are probably equally disabling in Bangladesh. Buildings in Bangladesh, both private and public, are not accessible for PWDs. The needs of PWDs are not considered during the design process [37]. Therefore, it is only when a household member becomes disabled the importance of an accessible home becomes obvious. However, perhaps, even the high income group participants were unable or unwilling to bear the costs of modifications of their buildings, as were the comparatively poorest group who were living in non-brick houses. Perhaps because of the same reasons, no evidence of an association was found between the severity of LD and living in one’s own or rented house.

Though area of residence has an association with the prevalence of disability in Bangladesh [34], no evidence of such association was observed between the severity of LD and the area of residence in this study. Urban areas in Bangladesh have better road networks than rural areas, but these roads are overcrowded with vehicles and people, and often without, or only with poorly conditioned, footpaths. Thus, perhaps people with LDs have to encounter an equally disabling outside world in both rural and urban Bangladesh.

This study findings need to be interpreted with caution since the participants were selected through a rehabilitation centre. This centre was the only specialised LD rehabilitation centre of its kind in Bangladesh [18]. But, an inequality in accessing LD rehabilitation services was observed when the socio-demographic characteristics of the participants were compared with those of the national averages. Literacy rate of this study participants’ was higher than those of the national rate [38]. Even though there were lost household income opportunities because of the presence of a disabled household member, the household income was higher among the study participants than the national average [27]. The per capita household income was also higher among the study participants than those of the national average [26]. The housing condition of the study participants was also better than the general housing standard in Bangladesh [27]. Only one third (32.3%) of the study participants were female, whereas, according to the national census, in 2011 the proportion of female was 49.9% in Bangladesh [38]. In addition, a previous survey reported that the prevalence of disability was higher among females [26]. A WHO survey also reported that the estimated world prevalence of disability was higher among women [15].

## Conclusions

Although socio-demographic variables are widely known to have an association with the presence of any disability, this study did not find any association of such variables with the severity of LD apart from age, occupation and income status. Perhaps, irrespective of their socio-demographic characteristics PWDs face the similar disabling built and attitudinal environments in Bangladesh. People with higher socioeconomic status might live in better quality houses, but those houses not necessarily are accessible for PWDs. This lack of association between the severity of LD and the majority of the demographic and socioeconomic position variables is believed to be the indication of the ineffectiveness of trying to fit PWDs in an existing non-inclusive society. This study results call for a state level involvement in making the services and built facilities inclusive in order to allow PWDs to live a better quality life.

## Additional file

Additional file 1: Table S1. The Locomotor Disability Scale (LDS) items. (DOCX 13 kb)

## Abbreviations

BDT: Bangladeshi currency
CBR: Community-based rehabilitation
CI: Confidence interval
CRP: Centre for the rehabilitation of the paralysed
ICF: International classification of functioning, disability and health
LD: Locomotor disability
LDS: Locomotor disability scale
PWDs: People with disabilities
SCI: Spinal cord injury
USD: United States dollar
WHO: World Health Organisation
