# Supplementary material for: Socio-demographic determinants of the severity of locomotor disability among adults in Bangladesh: a cross-sectional study, December 2010–February 2011
Source: Arch Public Health. 2017 Nov 20;75:47. doi: 10.1186/s13690-017-0217-5 (PMC5694906; doi:10.1186/s13690-017-0217-5)
Supplement: Additional file 1: Table S1. — The Locomotor Disability Scale (LDS) items. (DOCX 13 kb) [file 13690_2017_217_MOESM1_ESM.docx]

Additional file 1: Table S1. The Locomotor Disability Scale (LDS) items

| **Items** | **Score (0-4)** |
| --- | --- |
| 1. Sitting up from lying down |  |
| 1. Lying down from sitting up |  |
| 1. Standing up from sitting on a chair |  |
| 1. Sitting down on a chair |  |
| 1. Sitting down on the floor |  |
| 1. Standing up/sitting up on a wheelchair from a sitting position on the floor |  |
| 1. Getting into a squatting position |  |
| 1. Getting out of a squatting position |  |
| 1. Bending down and sideways |  |
| 1. Lifting objects |  |
| 1. Reaching for overhead objects |  |
| 1. Transferring oneself from a sitting position to another sitting position |  |
| 1. Maintaining a lying position |  |
| 1. Maintaining a squatting position |  |
| 1. Maintaining a sitting position on a chair |  |
| 1. Maintaining a sitting position on the floor |  |
| 1. Maintaining a standing position |  |
| 1. Walking inside the home |  |
| 1. Walking in the neighbourhood |  |
| 1. Walking on different surfaces |  |
| 1. Walking around obstacles |  |
| 1. Climbing up and down two flights of stair |  |
| 1. Walking in quick steps |  |
| 1. Jumping |  |
| 1. Carrying objects |  |
| 1. Getting into and out of own home |  |
| 1. Travelling by non motorised vehicles |  |
| 1. Travelling by private motorised vehicles |  |
| 1. Travelling by public transports |  |
| 1. Washing parts of body |  |
| 1. Taking a bath or shower |  |
| 1. Grooming |  |
| 1. Toileting |  |
| 1. Dressing |  |
| 1. Feeding |  |
| 1. Maintaining own health |  |
| 1. Praying |  |
| 1. Attending Ceremonies |  |
| 1. Shopping |  |
| 1. Washing and drying clothes and garments |  |
| 1. Cleaning living area |  |
| 1. Disposing of household garbage |  |
| 1. Maintaining dwelling and furnishings |  |
| 1. Assisting household members with self-care |  |
| 1. Assisting household members in movement |  |
| 1. Assisting household members in health maintenance |  |
| 1. Accessing public services |  |
| 1. Maintaining an occupation |  |
| 1. Doing voluntary social work |  |
| 1. Socialising |  |
| 1. Playing indoor games |  |
| 1. Attending arts, cultural and sports events |  |
| 1. Travelling for pleasure |  |
| 1. Gardening |  |
| 1. Watching television |  |
